# Childhood carcinoid tumors: description of a case series in a Brazilian cancer center

**DOI:** 10.1590/S1516-31802006000100005

**Published:** 2006-01-05

**Authors:** Gustavo Ribeiro Neves, Paulo Chapchap, Simone Treiger Sredni, Cristiano Ribeiro Viana, Wellington Luiz Mendes

**Keywords:** Carcinoid tumor, Neuroendocrine tumors, Diagnosis, Pathology, Surgery, Tumor carcinóide, Tumores neuroendócrinos, Diagnóstico, Patologia, Cirurgia

## Abstract

**CONTEXT AND OBJECTIVE:**

Carcinoid tumors are very rare both in children and adults. About 85% of these tumors develop in the gastrointestinal tract. The objective of the present study was to describe our experience with children treated of carcinoid tumors, and investigate the frequency morphological findings and results.

**DESIGN AND SETTING:**

Report on case series, at the Department of Pediatrics of Centro de Tratamento e Pesquisa Hospital do Câncer, São Paulo.

**METHODS:**

This was a retrospective analysis of clinical pathological data and outcomes among children (< 18 years old) with carcinoid tumors admitted from January 1, 1990, to December 31, 2001.

**RESULTS:**

Nine patients (mean age 12.2 years) were included: six girls and three boys (2:1), all of them Caucasian. In eight cases (89%), the primary tumor site was the appendix and in one (11%) it was the left bronchus. For those with primary tumor in the appendix, the main complaint was abdominal pain, which led to appendectomy. Only one patient underwent right hemicolectomy due to tumor extension into the serosa. The patient with bronchial tumor underwent left pneumonectomy. All patients had localized disease and are alive and free of disease. They have had follow-ups lasting from 1 to 11 years (mean of 3.5 years).

**CONCLUSION:**

Although the majority of carcinoid tumors arise from the appendix, these tumors can also occur in other primary sites. Surgical resection at an early stage allows for good prognosis without the need for any adjuvant treatment.

## RESUMO

**CONTEXTO E OBJETIVO:**

Tumores carcinóides são bastante raros, tanto em crianças quanto em adultos. Cerca de 85% deles desenvolvem-se no trato gastrintestinal. O objetivo do presente estudo foi descrever a experiência de nossa instituição com crianças tratadas de tumores carcinóides, investigando sua freqüência, achados morfológicos e resultados.

**TIPO DE ESTUDO E LOCAL:**

Relato de série de casos, no Departmento de Pediatria do Centro de Tratamento e Pesquisa Hospital do Câncer, São Paulo, Brasil.

**MÉTODOS:**

Análise retrospectiva de dados clinicopatológicos e resultados de crianças menores de 18 anos de idade com tumores carcinóides, admitidas entre 1° de janeiro de 1990 e 31 de dezembro de 2001.

**RESULTADOS:**

O estudo inclui nove pacientes, todos brancos, com idade média de 12,2 anos, sendo seis meninas e três meninos (2:1). O apêndice foi o sítio primário em oito casos (89%) e o brônquio esquerdo em um caso (11%). Nos primeiros, o sintoma principal foi dor abdominal que levou a apendicectomia. Apenas um paciente foi submetido a hemicolectomia direita em razão da extensão do tumor na serosa. O paciente que tinha o tumor primário no brônquio foi submetido a pneumectomia à esquerda. Todos os pacientes tinham doença localizada e estão vivos sem doença, com acompanhamento de 1 a 11 anos (média de 3,5 anos).

**CONCLUSÃO:**

Embora a maioria dos tumores carcinóides tenha sua origem no apêndice, eles podem ocorrer em outros sítios. A ressecção cirúrgica em estágios iniciais permite um bom prognóstico sem necessidade de tratamento adjuvante.

## INTRODUCTION

Carcinoid tumors are very rare both in children and adults. About 85% of these tumors develop in the gastrointestinal tract.^1^ Gastrointestinal carcinoid tumors are most commonly found in the appendix or ileum^2,3^ but they have been described in other locations such as in the gastrointestinal tract, lungs, thymus, and gonads.^2,4^

Carcinoid tumors are the most common neoplasms of the gastrointestinal tract found by physicians^5,6^ and their discovery is usually incidental during the course of another pro- cedure.^7^ In general, children are admitted to a hospital after complaining about pain in the lower abdominal quadrant, along with symptoms of acute appendicitis, and the diagnosis is performed after appendectomy and anatomopathological examination. Surgery remains the most important treatment type for patients treated with curative intention.^1,3^ The clinical course for patients diagnosed with gastrointestinal carcinoid tumors depends predominantly on the stage of the disease at diagnosis.^3,8^

Well-differentiated neuroendocrine tumors or carcinoids include a spectrum of neoplasms originating from the neuroendocrine system. In the small intestine, they seem to arise from intraepithelial endocrine cells, whereas appendix carcinoids seem to develop from subepithelial endocrine cells. Their malignancy depends on the size, depth and site of the tumor.^6,7,9^ These tumors are frequently discovered incidentally and measure < 2 cm in diameter.^6,7,10,11^

There are a number of substances that are secreted alone or in combination by these neoplasms, including 5-serotonin (5-HT), 5- hydroxytryptophan (5-HTP), kallikrein, adrenocorticotropic hormone (ACTH), histamine, P substance, prostaglandin, catecholamines and gastrin.^12,13^ These vasoactive substances produced by tumor cells cause carcinoid syndrome that presents with vasomotor flushing, episodic hypotension and diarrhea. Because these substances are metabolized during their first passage through the liver, gastrointestinal carcinoids do not cause carcinoid syndrome unless they are metastatic to the liver.^7,14^

Bronchial carcinoids account for 1% to 5% of all lung tumors. Bronchial obstruction is the cause of most of the signs and symptoms present, including cough, hemoptysis, obstructive pneumonitis, pleuritic pain, atelectasis and dyspnea.^15^ The association between bronchial carcinoid tumors and carcinoid syndromes is rare.

This retrospective study was conducted to determine the clinical pathological characteristics of carcinoid tumors in children at the Centro de Tratamento e Pesquisa (Treatment and Research Center) of Hospital do Câncer, São Paulo, Brazil, over the past 15 years, in order to better understand the natural history of this rare neoplasm.

## PATIENTS AND METHODS

All patients aged 18 years or less who underwent evaluation at our institution and were diagnosed with carcinoid tumors between January 1, 1990, and December 31, 2001, were identified through the files in the institution's cancer registry. The medical records were reviewed for data collection. The demographic data collected included gender, race, age at diagnosis and duration of followup. The anatomopathological review of all the cases was done by a pediatric pathologist in order to ensure data accuracy. The pathological diagnosis was confirmed in each case using established histological criteria.^16^ All cases were evaluated for number of mitosis per 10 highpower fields, nuclear pleomorphism, depth of invasion and immunohistochemical findings, when possible. Tumor size was obtained from the greatest diameter measured in the slide for all appendix tumors. Because our hospital is a cancer treatment center, all patients had been referred from other hospitals following surgery, except for patient number 9, who had a bronchial tumor that was surgically treated at our institution.

The length of follow-up was calculated from the date of diagnosis until the last clinical information on the patient up to December 2001.

## RESULTS

From January 1990 to December 2001, nine of the 4,656 (0.19%) children evaluated for malignancy at our institution were diagnosed with carcinoid tumors. Table 1 shows their epidemiological and clinical characteristics, and their treatment type. The patients’ ages at admission were 4-17 years (mean of 12.2 years). Six patients were girls, three were boys and all of them were white.

**Table 1:** Clinical characteristics, treatment and follow-up of nine pediatric patients with carcinoid tumors*

Patient	Sex	Age (years)	Main complaint	Site	Treatment	Date of diagnosis
1	M	9	Acute abdominal pain	Appendix	Appendectomy	Jul 31, 1990
2	F	11	Insidious abdominal pain for 6 months + acute abdomen	Appendix	Appendectomy	Jan 13, 1994
3	F	14	Insidious abdominal pain for 17 days + acute abdomen	Appendix	Appendectomy	Apr 22, 1998
4	F	13	Insidious abdominal pain for 6 months + acute abdomen	Appendix	Appendectomy	Jul 29, 1999
5	F	15	Insidious abdominal pain for 15 days + acute abdomen	Appendix	Appendectomy	Apr 3, 2000
6	M	17	Acute abdomen	Appendix	Appendectomy + hemicolectomy	Feb 28, 2001
7	F	4	Acute abdomen	Appendix	Appendectomy	Dec 17, 2001
8	F	15	Acute abdomen	Appendix	Appendectomy	Dec 9, 1999
9	M	12	Fever for 30 days	Bronchus	Left pneumonectomy	Aug 21, 2000

*M = male; F = female;*

* *all patients were alive and disease-free one to eleven years after surgery.*

**Figure 1:**
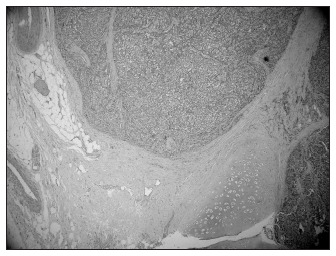
Bronchial carcinoid tumor (he- matoxylin-eosin; 40 x) of patient number 9, a 12-year-old boy.

In eight patients, the site of the carcinoid tumor was the appendix. One patient arrived at our hospital with persistent fever. A bronchial tumor mass was seen in the chest X-ray in the left side and biopsy revealed a carcinoid tumor (patient 9) (Figure 1).

Among the symptomatic patients with appendix tumors, in four cases the main complaint was abdominal pain and the initial diagnosis was appendicitis. In the other four cases, the initial diagnosis was acute abdomen. Symptoms of carcinoid syndrome (flushing or diarrhea) or Cushing's syndrome^7,17^ were not described in any of these patients.

Seven patients were submitted to appendectomy alone and one had a right hemicolectomy due to tumor invasion into the serosa and periappendiceal fat (patient 6), which is unusual in children. The patient with the bronchial tumor underwent pneumonectomy (patient 9). None of our patients received chemotherapy. The diagnosis was done through anatomopathological examination after surgery (Table 2).

**Table 2:** Pathological characteristics (tumor size, invasion, mitotic index and immunohistochemistry) of nine pediatric patients with carcinoid tumors

Patient	Tumor size (slide)	Depth of invasiveness	Mitotic index	Immunohistochemistry
1	0.5 cm	Well-delimited, without invasion	0/10	ND
2	0.8 cm	Serosa	1/10	ND
3	0.9 cm	Serosa	2/10	NSE + cytokeratin AE1/AE3 + chromogranin inconclusive
4	0.3 cm	Serosa	1/10	NSE + cytokeratin AE1/AE3 + chromogranin +
5	1.5 cm	Submucosa	1/10	NSE + cytokeratin AE1/AE3 + chromogranin +
6	1.7 cm	Serosa, infiltrating periappendiceal fat	11/10	ND
7	1.5 cm	Submucosa	0/10	ND
8	0.5 cm	Tunica muscularis	0/10	ND
9	3.5 × 2.5 × 2.0 cm*		1/10	ND

*ND = not done; NSE = neuron-specific enolase; + = positive.*

* *Patient number 9 was the only one who underwent surgery at our institution and therefore this patient was the only case in which tumor measurements were made on the surgical specimen.*

**Figure 2:**
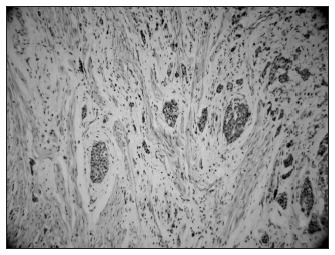
Carcinoid tumor in the appendix, showing clusters of tumor cells in tunica muscularis (hematoxylin-eosin; 100 x) of patient number 8, a 15-year-old girl.

Anatomopathological examination showed small, uniformly stained clear cells with moderate amounts of finely granular cytoplasm arranged in solid, trabecular, insular, and organoid patterns with tiny islands of tumor cells surrounded by thin layers of stroma. The tumor size was quite small in most cases, and was 1 cm or less in all appendix specimens (0.15 cm to 1.7 cm; mean of 0.79 cm). Analysis of tumor invasiveness showed that only one tumor remained restricted to the appendix mucosa (patient 5). Three tumors invaded the serosa (patients 2, 3 and 4), one invaded the muscularis (patient 8; Figure 2) and one invaded the periappendiceal fat (patient 6). Patient 6 underwent right hemicolectomy as an additional procedure.

In no case was residual or metastatic disease identified.

All patients were alive and free of disease, up to eleven years after surgery, considering December 2001 as the end of follow-up for this study (mean follow-up duration of 3.5 years).

## DISCUSSION

Carcinoid tumors in children younger than 18 years old accounted for 0.19% of all pediatric cases admitted to our institution during the study period. Spunt et al. reported on eight cases out of a total of 10,138 patients (0.08%) treated over a 22-year period at a pediatric oncohematology hospital.^9^ Doede et al. reported on eight cases of children with carcinoid tumors out of a total of 4,747 (0.169%) appendectomies performed over a 12-year period.^18^ These data illustrate the rarity of carcinoid tumors and explain the low rate of suspicion of this disease. In other pediatric series, the diagnosis was made following appendectomy.^6,7,18^

In our study, the appendix was the most common primary site, accounting for eight out of our nine cases. The patients in our series were older children and adolescents, with a mean age of 12.2 years at presentation, and there was female predominance (2:1), although there does not seem to be consistent gender preference among adult cases. Our data are similar to those in another pediatric series, where the female predominance was 3:1, the median age was 12.7 years, and the primary site was the appendix.^9^

No metastatic tumor was found in any case, as also found in other pediatric series. This illustrates the low malignant potential of carcinoid tumors in children. Volpe et al. reported on a patient with carcinoid tumor from the appendix with one metastatic lymph node, out of 50 others analyzed.^14^ Spunt et al. found liver metastasis from a primary tumor of the ovary.^9^ Although metastases are present at diagnosis in approximately one third of adults with carcinoid tumors (commonly involving lymph nodes, liver, lung, mesentery and bones), metastatic disease at diagnosis rarely is associated with tumors of appendix origin^19^, except if the tumors are 2 cm or larger in diameter.^20^

In the literature on adult populations, it has been reported that tumor size larger than 2 cm, deep invasion and lymph node involvement are poor prognostic factors.^20^ In such cases, the surgeon would be obliged to remove all the tissue involved, the primary lymphatic drainage route and the right hemicolon.^7^ Corpron et al. questioned the real need for such an aggressive procedure when treating children.^6^ These authors observed adult series and compared the mortality related to metastatic disease and to tumors larger than 2 cm that were treated with appendectomy or right hemicolectomy, and found no additional benefits from the latter surgical approach. In another two substantial studies, no patients with tumors smaller than 2 cm developed metastasis or local relapse over a follow-up period of 11-28 years.^10,21^ Our experience showed one child who underwent right hemicolectomy due to tumor invasion of the appendix serosa.

Carcinoid syndrome is rarely found in pediatric patients, although these phenomena have been described in cases of carcinoid tumors of the appendix in other reports.^7^ The symptoms of carcinoid syndrome are caused by vasoactive substances such as 5-hydrotryp- tophan (5-HIAA), histamine, bradykinin and serotonin that are produced by tumor cells. Because these substances are metabolized during their first passage through the liver, gastrointestinal carcinoids do not cause carcinoid syndrome unless they are metastatic to the liver and ovary.^14,22,23^ In our data, 5-HIAA measurements from urine samples were only made for two patients, 30 and 42 days after surgery. These showed normal levels of this serotonin metabolite, perhaps due to the long time that had elapsed following the surgery. High levels of 5-HIAA have been associated with large-volume tumors.^14^ Spunt et al. described high levels only in one of the eight patients who had metastatic disease.^9^

Masson et al.^24^ were the first to demonstrate that appendix carcinoids are derived from a proliferation of Kultschitzky cells. Neuroendocrine cells related to these enterochromaffin cells that were found in the gastrointestinal tract and associated with carcinoid tumors were subsequently found in the bronchopulmonary tree. There, where they form part of a histological spectrum that includes small cell lesions (which have the most malignant biological behavior), typical carcinoids (which have the most benign behavior) and atypical carcinoids.^25^ Approximately 90% of carcinoid tumors are typical tumors.^26^ These are predominant in pediatric series and present favorable prognosis. Atypical carcinoid tumors are rare and much more difficult to diagnose histologically. They present worse prognosis than typical lesions, with high metastatic potential, showing increased mitotic activity, pleomorphism with hyperchromatic nuclei, abnormal ratio of nucleus to cytoplasm, increased cellularity, disorganization and necrosis.^27^

An association between carcinoid tumors and colonic adenocarcinoma has been reported,^28^ although such an association in childhood is extremely rare. Carcinoid tumors in adults have shown a correlation between pathological behavior and primary site. Levi et al. reported on a series of 433 adult patients in which 174 (40%) presented colorectal tumors, followed in frequency by small intestine (27%), lung (18%) and stomach tumors (4%). The lungs were the most common site for malignant carcinoids, and the intestines (mainly the colorectum) for benign ones; less than 5% of benign carcinoids were outside the digestive tract. Overall, 141 (81%) out of 174 colorectal tumors, and 14 (74%) out of 19 gastric tumors were be- nign.^26^ The prevalence of benign tumors in the small intestine and lungs was 38% and 6%, respectively. With these findings, the question posed is whether the good prognosis for carcinoid tumors in childhood is due to their primary site of occurrence (appendix) or to their benign pathological behavior in the majority of cases.

The only patient with a bronchial tumor in our series had no specific symptomatology and underwent pneumonectomy. Fink et al.^15^ reported on 142 adult patients, of whom 62% had tumors localized in the main bronchus. The symptoms included cough and airway obstruction. A surgical approach was adopted in 79% of the cases. Even though we did not find associated synchronous or metachronous neoplasms in our group of patients, other authors have reported the presence of colonic adenocarcinoma and also higher chances of cardiovascular diseases.^1,9^ Gastrointestinal carcinoid disease is rather uncommon. Therefore, a diagnosis of gastrointestinal carcinoid tumors with or without metastasis is frequently an incidental intraoperative finding.

Although the majority of our patients had primary carcinoid tumors in the appendix, these tumors can occur at other sites. Early diagnosis may be an important factor for preventing morbidity and mortality, since patients with localized disease have better outcome, whereas metastatic disease indicates poor prognosis.

Appendectomy is the therapy of choice. More aggressive surgical treatment with cecal or ileocecal resection may be indicated for treating large tumors. Right hemicolectomy should be limited to restricted cases and surgery should be performed only when either the line of resection is not tumor-free or the tumor has already metastasized to local lymph nodes. Although curative therapy has not yet been defined for metastatic patients, octreotide and its synthetic analogs may reduce the symptoms of carcinoid syndrome.^29^

## CONCLUSIONS

In childhood, carcinoid tumors are neuroendocrine tumors that are most commonly found in the appendix, although they can occur in other sites. Our retrospective experience showed that, when early diagnosed and treated, these patients may present good prognosis and low probability of metastatic lesions. Appendectomy alone is usually the treatment of choice and long-term survival is almost universal in these cases.

## References

[1] Soreide JA, van Heerden JA, Thompson GB (2000). Gastrointestinal carcinoid tumors: long-term prognosis for surgically treated patients. World J Surg.

[2] Agranovich AL, Anderson GH, Manji M, Acker BD, Macdonald WC, Threlfall WJ (1991). Carcinoid tumour of the gastrointestinal tract: prognostic factors and disease outcome. J Surg Oncol.

[3] Shebani KO, Souba WW, Finkelstein DM (1999). Prognosis and survival in patients with gastrointestinal tract carcinoid tumors. Ann Surg.

[4] Olney JR, Urdaneta LF, Al-Jurf AS, Jochimsen PR, Shirazi SS (1985). Carcinoid tumors of the gastrointestinal tract. Am Surg.

[5] Moertel CG, Dockerty MB, Judd ES (1968). Carcinoid tumors of the vermiform appendix. Cancer.

[6] Corpron CA, Black CT, Herzog CE, Sellin RV, Lally KP, Andrassy RJ (1995). A half century of experience with carcinoid tumors in children. Am J Surg.

[7] Moertel CL, Weiland LH, Telander RL (1990). Carcinoid tumor of the appendix in the first two decades of life. J Pediatr Surg.

[8] McDermott EW, Guduric B, Brennan MF (1994). Prognostic variables in patients with gastrointestinal carcinoid tumours. Br J Surg.

[9] Spunt SL, Pratt CB, Rao BN (2000). Childhood carcinoid tumors: the St Jude Children’s Research Hospital experience. J Pediatr Surg.

[10] Svendsen LB, Bulow S (1980). Carcinoid tumours of the appendix in young patients. Acta Chir Scand.

[11] Assadi M, Kubiak R, Kaiser G (2002). Appendiceal carcinoid tumors in children: does size matter?. Med Pediatr Oncol.

[12] Creutzfeldt W, Stockmann F (1987). Carcinoids and carcinoid syndrome. Am J Med.

[13] Eriksson B, Oberg K (1991). Peptide hormones as tumor markers in neuroendocrine gastrointestinal tumors. Acta Oncol.

[14] Volpe A, Willert J, Ihnken K, Treynor E, Moss RL (2000). Metastatic appendiceal carcinoid tumor in a child. Med Pediatr Oncol.

[15] Fink G, Krelbaum T, Yellin A (2001). Pulmonary carcinoid: presentation, diagnosis, and outcome in 142 cases in Israel and review of 640 cases from the literature. Chest.

[16] Rosai J, Rosai J (1996). Gastrointestinal tract. Ackeman surgical pathology.

[17] Moertel CG (1987). Karnofsky memorial lecture. An odyssey in the land of small tumors. J Clin Oncol.

[18] Doede T, Foss HD, Waldschmidt J (2000). Carcinoid tumors of the appendix in children -epidemiology, clinical aspects and procedure. Eur J Pediatr Surg.

[19] Soga J (1998). Statistical evaluation of 2001 carcinoid cases with metastases, collected from literature: a comparative study between ordinary carcinoids and atypical varieties. J Exp Clin Cancer Res.

[20] Moertel CG, Weiland LH, Nagorney DM, Dockerty MB (1987). Carcinoid tumor of the appendix: treatment and prognosis. N Engl J Med.

[21] Parkes SE, Muir KR, al Sheyyab M (1993). Carcinoid tumours of the appendix in children 1957-1986: incidence, treatment and outcome. Br J Surg.

[22] Wilkowske MA, Hartmann LC, Mullany CJ, Behrenbeck T, Kvols LK (1994). Progressive carcinoid heart disease after resection of primary ovarian carcinoid. Cancer.

[23] Vergani D, Massironi L, Lombardi F, Fiorentini C (1998). Carcinoid heart disease from ovarian primary presenting with acute pericarditis and biventricular failure. Heart.

[24] Masson P (1928). Carcinoids (argentaffin-cell tumors) and nerve hyperplasia of the appendicular mucosa. Am J Pathol.

[25] Arrigoni MG, Woolner LB, Bernatz PE (1972). Atypical carcinoid tumors of the lung. J Thorac Cardiovasc Surg.

[26] Levi F, Te VC, Randimbison L, Rindi G, La Vecchia C (2000). Epidemiology of carcinoid neoplasms in Vaud, Switzerland, 1974-97. Br J Cancer.

[27] Hulka GF, Rothschild MA, Warner BW, Bove KE (1996). Carcinoid tumor of the trachea in a pediatric patient. Otolaryngol Head Neck Surg.

[28] Rivadeneira DE, Tuckson WB, Naab T (1996). Increased incidence of second primary malignancy in patients with carcinoid tumors: case report and literature review. J Natl Med Assoc.

[29] Comaru-Schally AM, Schally AV (2005). A clinical overview of carcinoid tumors: perspectives for improvement in treatment using peptide analogs (review). Int J Oncol.

